# P-882. Comparison of Epidemiology, Risk factors, Antimicrobial susceptibility and Outcome of Anaerobic Bacteremia: 2011-2013 and 2020- 2022

**DOI:** 10.1093/ofid/ofae631.1073

**Published:** 2025-01-29

**Authors:** Sivanthini Palanivetpillai, Mao-Cheng Lee, Prenilla Naidu

**Affiliations:** University of Alberta, Edmonton, Alberta, Canada; University of Alberta, Edmonton, Alberta, Canada; University of Alberta, Edmonton, Alberta, Canada

## Abstract

**Background:**

Anaerobes are again entering the spotlight as patients become more complex and antimicrobial resistances continue to mount as a result of their widespread use. The aim of our study was to compare the incidence, risk factors and susceptibility patterns of anaerobic bacteremia during 1st Jan 2011-31st Dec 2013 (period 1) and 1st Jan 2020-31st Dec 2022 (period 2) in community hospitals and acute care centres in Edmonton and the North Zone in Alberta.

The percentages of anaerobic organisms isolated in 2011-2013 and 2020-2022

**Figure:**
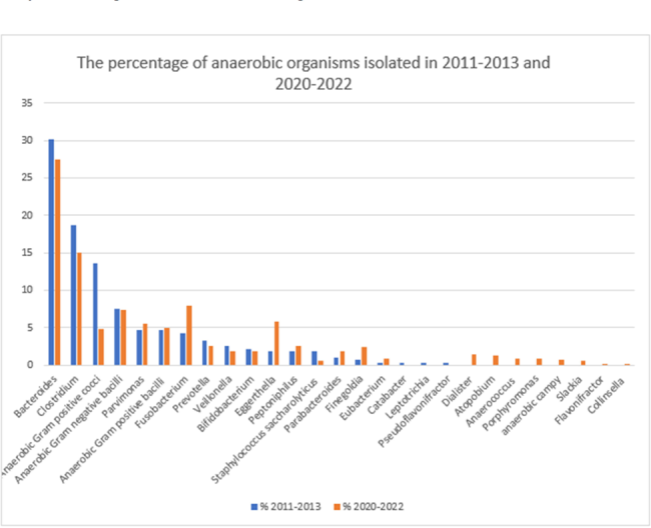

**Methods:**

• Microbiologic data extracted from laboratory information system

• Demographic and clinical data extracted from the EMR system

• Blood cultures were incubated on the BD BACTEC 9240™ system or the BD BACTEC™ FX Blood Culture System

• Organism identification was performed by Vitek® 2 ANC ID card, traditional phenotypic methods, Vitek® MALDI-TOF and, failing these, genomic sequencing

• Susceptibility testing by E-test; interpretation as per CLSI or EUCAST guidelines

The percentages of susceptible Bacteroides species to different antimicrobials in 2011-2013 and 2020-2022

**Figure:**
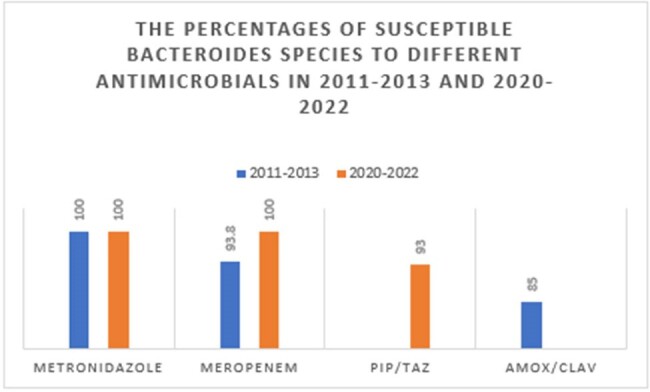

**Results:**

Period 1:

319 anaerobic bacteremia were identified. 247 were included in the study after exclusion of repeat positives (within the first 2 weeks of the first positive culture) and contamination. Total number of isolates were 279 and susceptibilities were available on 252 during this period.

Period 2:

830 anaerobic bacteremia were identified. 467 anaerobic bacteremia were included in the study after exclusion of repeat positives and contamination. The total number of isolates were 539 and susceptibilities were available on 417 isolates during this study period.

The total number of anaerobic bacteremia almost doubled from 82 cases per year to 156 cases per year during the study decade. The majority were monomicrobial bacteremia (71% and 85%) with the gastrointestinal tract identified as the commonest source. The most common isolated anaerobe was Bacteroides species. Metronidazole susceptibilities for all anaerobes were above 90%. The crude 30-day mortality rates were 22.7% and 25.8% in period 1 and 2 respectively.

**Conclusion:**

The total number of anaerobic bacteremia increased significantly in the 9-year interval. No significant changes in metronidazole susceptibility were observed between both periods of time.

**Disclosures:**

**All Authors**: No reported disclosures

